# Biofilm disruption and bactericidal activity of aqueous ozone coupled with ultrasonic dental scaling

**DOI:** 10.1016/j.jfscie.2021.100003

**Published:** 2022-02-08

**Authors:** Kevin C. Failor, Bruce Silver, Westin Yu, Jason E. Heindl

**Affiliations:** a Department of Biological Sciences, University of the Sciences in Philadelphia, Philadelphia, PA; b Silver Dental Associates, Burlington Township, NJ; c Lake Erie College of Osteopathic Medicine, Erie, PA

**Keywords:** Dental aerosols, biofilms, communicable disease control, dental equipment, microbial load

## Abstract

**Background:**

The COVID-19 pandemic has heightened the awareness of a common hazard encountered in the dental clinic: aerosol transmission of pathogens. Treatment of sources of infection before or during dental procedures is one means of decreasing pathogen load and aerosol transmission.

**Methods:**

An ultrasonic scaler supplied with aqueous ozone was used to examine the effect of its viability on planktonic cultures and biofilms formed by 2 model bacteria: *Rothia mucilaginosa* and *Escherichia coli*.

**Results:**

Both organisms showed susceptibility to aqueous ozone alone (97% and 99.5% lethality, respectively). When combined with manual scaling using an ultrasonic scaler, a greater than 99% reduction in colony-forming units (CFUs)/mL could be reached with an aqueous ozone concentration of approximately 2 mg/L (*R. mucilaginosa*) or 0.75 mg/L (*E. coli*) after 5 through 6 seconds of scaling.

**Conclusions:**

Aqueous ozone coupled with ultrasonic scaling exhibited a higher efficiency of microbial kill than either method used alone***.*** Both gram-positive and gram-negative species were affected by this treatment. Studies on other oral microbiota constituents, including fungi and viruses, will provide information on the efficacy of this method on a greater biological scale. Studies to verify concomitant reduction of microbial load in dispersed aerosols in clinical settings should be completed to support practical applications of this treatment.

**Why Is This Important?**

Aerosol production and transmission of pathogens via dental instruments and procedures is a concern for dental health care providers. To decrease microbial load and reduce unwanted microbial exposure to both patients and providers, several off-the-shelf chemical treatments have been applied to the instruments in the absence of controlled experiments or data supporting their utility. Ozone is a strong oxidant known to kill bacteria and reduce microbial load and has been used in several clinical settings in pre- or posttreatment applications. The authors confirmed the bactericidal activity of aqueous ozone at concentrations easily achievable in the dental clinic. In addition, the authors combined aqueous ozone with ultrasonic dental scaling and showed that this combination had greater bactericidal activity than either aqueous ozone or dental scaling alone. Using aqueous ozone in ultrasonics for dental handpieces may aid in the reduction of aerosolized pathogens.

## Introduction

Dental handpieces and ultrasonic procedures have been identified previously as contributing large quantities of airborne particulates, microorganisms, and viruses into the local environment via aerosols.^1^, ^2^, ^3^ These aerosols are capable of spreading nearly 2 m from the operative site during ultrasonic scaling, resulting in high microbial contamination of the surrounding surfaces and the dental health care provider.^2^ With the emergence of severe acute respiratory syndrome coronavirus 2 (SARS-CoV-2), the control of the spread of aerosolized pathogens in dentistry has again come to the forefront. The use of polymer additives such as polyacrylic acid or xanthan gum to reduce or eliminate aerosol production is one potential method, although it does not eliminate pathogens.^4^ An alternative option is to use a sterilization method in tandem with dental procedures to reduce the aerosolized pathogen load. Several preprocedural mouthrinses, including aqueous ozone, have been investigated previously and have shown a reduction in surface contamination,^5^ although the concurrent use of these compounds with ultrasonic scaling has not been examined.

The human oral cavity is a highly diverse microbiome and is host to hundreds of bacterial species, each with its own environmental and physiological importance.^6^, ^7^ Many of these bacteria reside in the dental plaque biofilm, a functionally and structurally organized commensal community interlinked via a meshlike substrate of secreted, extracellular polymeric substances.^8^ These plaques form in a predictable, ordered fashion but comprise a highly variable microbial composition based on the physical location of each organism.^9^ One commonly isolated oral microflora, *Rothia mucilaginosa* (previously called *Micrococcus mucilaginosus* or *Stomatococcus mucilaginosus)* has been commonly identified from the oral cavity of both healthy individuals and patients with underlying conditions such as atherosclerosis, although it has been observed with a higher frequency in healthy populations.^10^, ^11^ In biofilms such as dental plaques, this gram-positive, encapsulated, nonmotile, bacterium forms multispecies microcolonies or, in the case of high-biomass regions, small islands^12^ and shows resilience even after multiple endodontic treatments.^13^ Although *R. mucilaginosa* is typically nonpathogenic in healthy individuals, it can act as an opportunistic pathogen and lead to conditions such as pneumonia,^14^ bacteremia,^15^ and endocarditis,^16^ and cause the buildup of biofilms on prosthetics,^17^ among others.^18^

Ultrasonic activation of aqueous ozone has been shown to be a means of disinfection of wastewater,^19^ and a combination of gaseous ozone and ultrasonic activation has been shown to be effective in disrupting biofilms of *Enterococcus faecalis* in the root canals of extracted teeth.^20^ Similarly, combination treatment using aqueous ozone and ultrasonic activation was effective against planktonic *Escherichia coli* cultures.^21^ Gaseous and aqueous ozone have been explored previously as potential agents for sanitizing dental instruments; however, their efficacy in disrupting dental plaques when used in tandem with an ultrasonic scaler has not been explored.^22^ These previous studies have shown the efficacy of ozone as a contact-sterilizing agent, but exposure times and efficacy limit the accessibility of the treatment for routine patient procedures.^22^ In this study, the opportunistic pathogen *R. mucilaginosa* and the gram-negative model *E. coli* were exposed to aqueous ozone in suspension and in an ultrasonic scaler after production of a monoculture biofilm. The data confirm the effectiveness of aqueous ozone against planktonic bacteria and extend these observations to the enhanced effectiveness of combining ultrasonic activation with aqueous ozone on bacterial biofilms.

## Methods

### Sterilization and sanitation

Before each experimental run, the ultrasonic scaler handpiece and tip, tweezers, water, and media were autoclaved to ensure proper sterilization. Materials that could not be sterilized (the aqueous ozone chamber and ultrasonic instrument reservoir) were sprayed and wiped down with 75% ethanol and allowed to dry fully. Before use, the aqueous ozone chamber, ultrasonic instrument reservoir, and all tubing was rinsed and flushed with sterile water to further ensure proper sanitization. Between individual runs, the ultrasonic scaler and tip and the tweezers were submerged in 75% isopropyl alcohol to limit cross-contamination. All 12- and 96-well plates, lids, and plastic coverslips were exposed to UV light for a minimum of 15 minutes before use.

### Bacteria

The M9 minimal media broth was prepared from a 5× solution supplemented with glycerol at a final concentration of 2% (vol/vol), without the addition of exogenous thiamine. The lysogeny broth (LB) agar plates were prepared following the standard recipe for the broth (10 g/L sodium chloride, 10 g/L Bacto tryptone (BD Biosciences), and 5 g/L yeast extract (BD Biosciences)) with 15 g/L agar. The brain-heart infusion (BHI) medium and agar plates were prepared using a Bacto reagent (BD Biosciences) and supplemented with agar when necessary. *E. coli* MG1655 and *R. mucilaginosa* 5762/67 were originally obtained from the American Type Culture Collection (47076 and 25296, respectively). Strains were grown initially on LB agar (*E. coli)* or BHI agar (*R. mucilaginosa*) overnight at 37 °C to obtain single colonies. Both organisms have been passaged repeatedly under laboratory conditions and, as a result, may have accumulated mutations altering their physiology from that described previously. One example of this is the loss of a requirement for exogenous thiamine when grown in M9 medium for *E. coli* MG1655.

## Static biofilm preparation

A previously described static biofilm assay for *Agrobacterium* species^23^ was adapted for use in this study. Overnight cultures of *E. coli* and *R. mucilaginosa* were grown at 37 °C in M9 and 2% (vol/vol) glycerol and BHI, respectively, and subcultured to an optical density (OD) of 0.1 at 600 nm (OD_600_). Cultures were grown at 37 °C with aeration until an OD_600_ of 0.4 through 0.6 was reached. The samples were then diluted to an OD_600_ of 0.05, and 3 mL were dispensed into each well of a sterile 12-well plate containing a vertically placed, sterile coverslip. All cultures were incubated in a humidified chamber for 48 hours at 37 °C. After incubation, the coverslips were washed vigorously 3 times with sterile water to remove loosely adherent cells and biomass. The remaining tightly adherent cells and biomass were then treated with ultrasonic scaling as described below.

## Experimental setup

Aqueous ozone was prepared using a method similar to the one outlined by César and colleagues.^22^ Pure oxygen was passed through an ozone generator (high power ozone generator machine Ozonator, [Dr O Solutions]) and bubbled into 750 mL of sterile Milli-Q water. Aqueous ozone concentrations were measured using the Vacu-vials ozone test kit (CHEMetrics K-7423). For the initial analysis of the effect of aqueous ozone on *R. mucilaginosa*, a minimum ozone concentration of 1.0 mg/L was used. Overnight cultures of both *E. coli* and *R. mucilaginosa* were subcultured to an OD_600_ of 0.01 and grown for 2 hours at 37 °C to ensure active growth. Five microcentrifuge tubes containing 1.0 mL of culture for each strain were spun at maximum speed (16,100*g*) for 2 minutes, aspirated, and washed with 1.0 mL of sterile water. This process was performed 3 times, and then the pelleted cells were stored at room temperature. The pellets were stored for a maximum of 4 hours before use. The pelleted cells were resuspended by vortexing after 1.0 mL of aqueous ozone was added to 4 tubes for each strain and 1.0 mL sterile water was added to the fifth. Two hundred μL of sample was aliquoted into 3 wells of a 96-well plate from the first tube (T_0_ being _time at which culture is first exposed to aqueous ozone_). Five 10-fold dilutions were prepared from the initial wells. One hundred μL of each dilution plated onto the LB media for *E. coli* and BHI for *R. mucilaginosa* and incubated overnight at 37 °C. The resulting colonies were counted to determine total colony-forming units (CFU) per mL.

**Figure 1:**
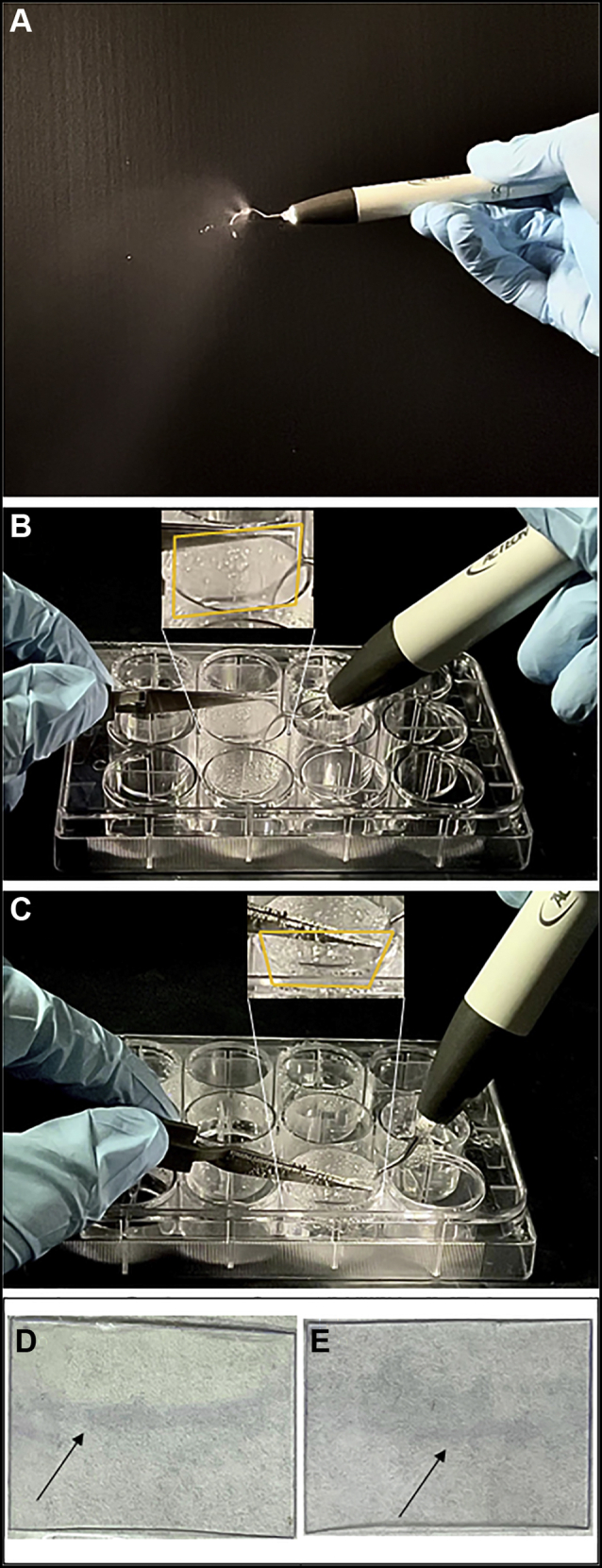
Ultrasonic scaler used for disruption of *Escherichia coli* and *Rothia mucilaginosa* biofilms. **A**. Dispersal pattern of activated ultrasonic scaler. **B**. Scaling of the front side of a biofilm-coated plastic coverslip in the sterile 12-well plate. **C**. Scaling of the back side of an alternate biofilm-coated plastic coverslip. Picture inserts in **B** and **C** highlight the approximate outline of the plastic coverslip being processed with an orange box. **D, E** are representative rinsed biofilms before treatment of *E. coli* and *R. mucilaginosa*, respectively (arrows point to adherent biomass).

An ultrasonic dental scaler (Newtron P5 XS B.LED; ACTEON) using either sterile water or aqueous ozone was used to disrupt 48-hour old–biofilms of *E. coli* MG1655 and *R. mucilaginosa* 5762/67. Aqueous ozone was prepared as detailed earlier until an ozone concentration greater than 4.0 mg/L was reached; 300 mL of aqueous ozone was transferred to the ultrasonic instrument reservoir. Using the ultrasonic scaler, water was dispensed for 2 through 3 seconds during the abrasion of coverslips to disrupt the biofilm (Figure 1A, B). This process was repeated on the opposite side to ensure maximum biofilm disruption (Figure 1C). Four coverslips were processed for each ozone concentration per run. New ozone concentrations were used every 15 through 20 minutes to account for the decomposition of the ozone and to mark the start of the next run. Target ozone concentrations were 4.00, 2.00, 1.00, and 0.50 mg/L. A separate run was performed using sterile water that was not exposed to any ozone and accounted for the less than 0.01 mg/L ozone concentration values. Six 10-fold dilutions were prepared for each sample. One hundred μL of each dilution was plated on LB or BHI and incubated overnight at 37 °C after which individual colonies were counted and CFUs/mL calculated.

### Statistical analysis

Bacterial concentrations were normalized to the sterile water-treated conditions of each strain for both sets of data before statistical analysis. For all biological variables, the mean values were compared first with an analysis of variance followed by the *t* test. Any differences and correlations were considered significant when the *P* values were scored below .05.

## Results

### Direct exposure of *E. coli* and *R. mucilaginosa* to aqueous ozone results in a greater than 97% reduction in CFUs/mL after 30 minutes

Gaseous and aqueous ozone have been shown previously to be effective at killing several bacterial genera pathogenic to humans, including *Staphylococcus*, *Streptococcus*, *and Escherichia*. Because of this interaction across both gram-positive and gram-negative species, it was predicted that a gram-positive *Rothia* species would be equally affected by this disinfecting agent.

**Figure 2:**
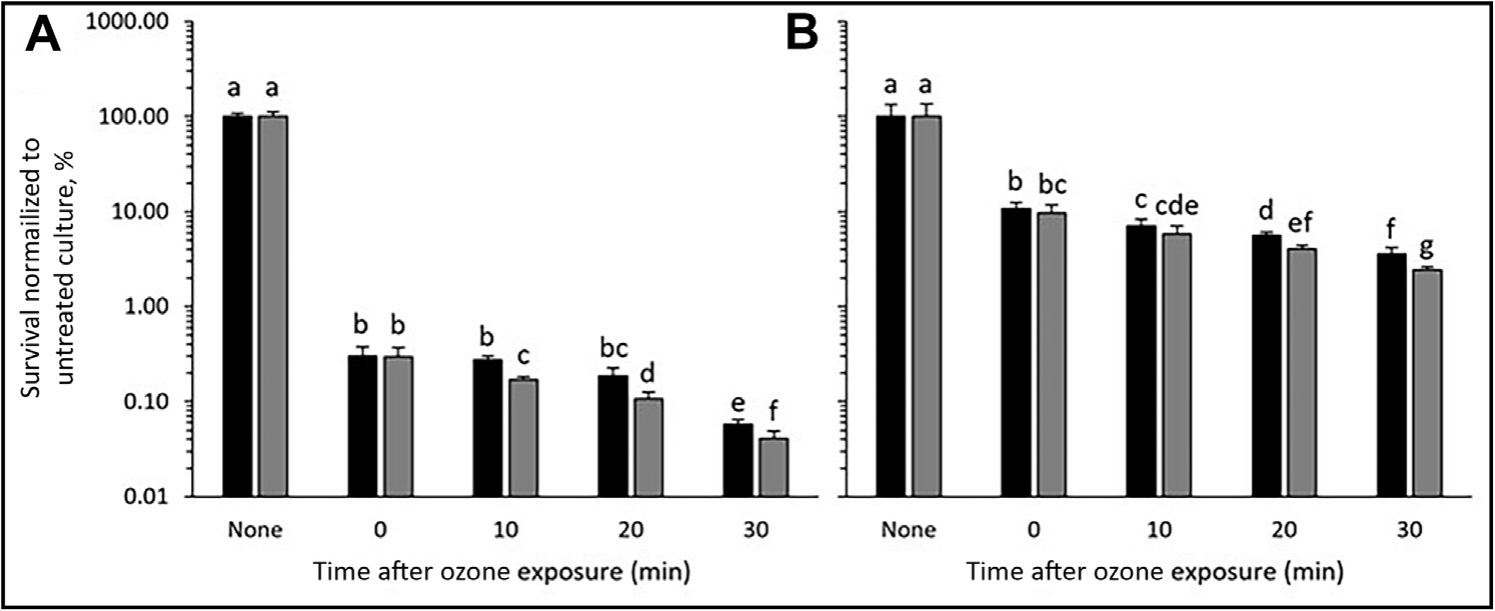
Effect of ozone on pure cultures of **A.***Escherichia coli* and **B.***Rothia mucilaginosa*. Cell suspensions of each bacterial species were exposed to aqueous ozone. Viable colony counts were performed every 10 minutes after exposure and compared with those of untreated samples (samples suspended in sterile water), listed here as None. Data were normalized to the mean untreated colony counts for each run. Black bars correspond to a starting ozone concentration of 1.19 mg/L and gray bars correspond to a starting ozone concentration of 1.38 mg/L. Bars with the same letter correspond to values that are not statistically different from one another via *t* test (*P* < .05). Error bars represent the mean (standard deviation) for each data point. 95% CIs for each data set are as follows (listed in order shown): **A**. Run 1: 9.0034, 0.0838, 0.0319, 0.0476, 0.0073; Run 2: 14.3241, 0.0834, 0.0162, 0.0199, 0.0092; **B**. Run 1: 38.1987, 1.8603, 1.4401, 0.5130, 0.6444; Run 2: 41.3413, 2.4249, 1.3701, 0.4052, 0.2302.

To test this, pure cultures of the previously studied *E. coli* and the organism of interest, *R. mucilaginosa*, were exposed to aqueous ozone at a concentration greater than 1.0 mg/L. The survivability of the culture was examined every 10 minutes and compared with that of a sample that had been resuspended in nonozonated water (Figure 2). These data show that *E. coli* is highly susceptible to the effects of aqueous ozone, resulting in a statistically significant 99.70% (standard deviation [SD] 0.07%) decrease in CFU formation immediately after the exposure (T_0_) and a 99.95% (SD 0.01%) decrease after 30 minutes of exposure (Figure 2A). The data for *R. mucilaginosa*, however, show a higher resistance to the lethal effects of ozone, with only an 89.74% (SD 1.82%) decrease in CFUs/mL after the initial T_0_ exposure, although this difference remains significant compared with the untreated values. Likewise, the data after 30 minutes show an increased resistance, resulting in a 96.99% (SD 0.74%) decrease in CFUs/mL (Figure 2B) suggesting that *R. mucilaginosa* is more resistant to ozone exposure or better adapted for handling oxidative stress.

### Combination of ultrasonic scaling and aqueous ozone amplifies the efficacy of biofilm disruption

The formation of bacterial biofilms poses an increased risk owing to the inherent difficulty in inhibiting or killing the bacteria present within the biofilm.^8^^,^^9^ While dental plaques are not a significant health concern, some oral microbes, including *Rothia* can form biofilms elsewhere in the body. In the light of this, a second assay was performed to determine the efficacy of disrupting *R. mucilaginosa* and *E. coli* biofilms using aqueous ozone and an ultrasonic scaler.

**Figure 3:**
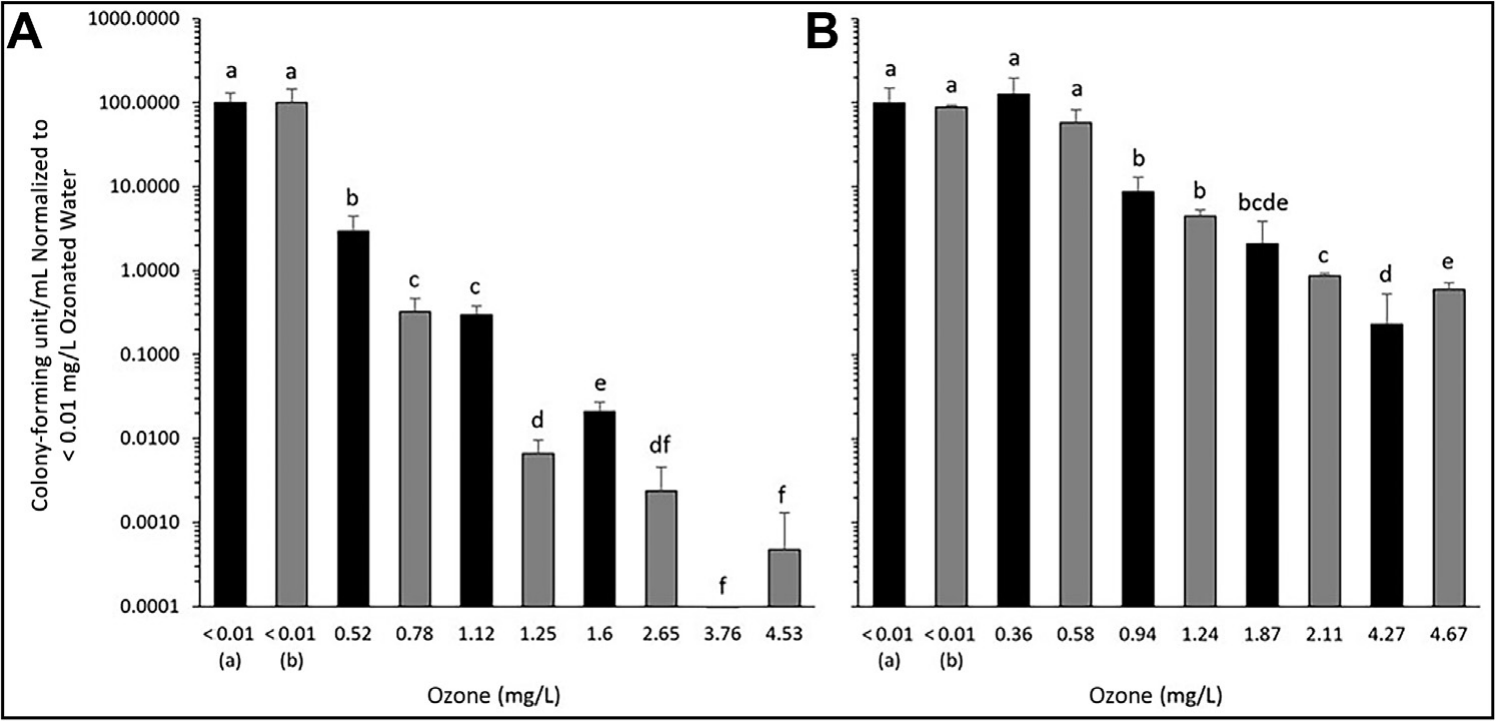
Effect of manual scaling using an ultrasonic scaler paired with varying concentration of aqueous ozone on pure cultures of **A**. *Escherichia coli* and **B**. *Rothia mucilaginosa*. Forty-eight-hour-old biofilms of each bacterial species were disrupted for 2 through 3 seconds on each side of the plastic coverslip using the activated ultrasonic scaler. Viable colony counts were performed using the resulting cell suspension and compared with suspensions prepared with sterile water. Data were normalized to the mean sterile water preparation colony counts for each experimental setup. Black bars correspond to Run 1 and gray bars correspond to Run 2. Bars with the same letter correspond to values that are not statistically different from one another via *t* test (*P* < .05). Error bars represent the mean (standard deviation) for each data point. 95% CIs for each data set are as follows (listed in order shown): **A**. 27.8402, 1.7237, 0.1683, 0.0873, 0.0034, 0.0068, 0.0025, null, 0.0009; **B**. 26.1199, 78.5414, 27.2605, 4.8902, 0.9053, 2.0138, 0.0838, 0.3364, 0.1322.

After 5 through 6 seconds of scaling using the ultrasonic scaler and nonozonated water, 1,950.00 (SD 593.55 CFUs/mL) (Run 1) and 700.00 (SD 320.78 CFUs/mL) (Run 2) were observed. The addition of ozone significantly decreased the observed CFUs/mL in each sample with an ozone concentration of 0.52 mg/L resulting in a 97.06% (SD 1.52%) decrease in CFUs/mL and a concentration of 4.53 mg/L resulting in a 99.9995% (SD 0.0008%) decrease (Figure 3A, Table).

**Table:** Raw and normalized descriptive statistics of the colony-forming units (CFUs)/mL for *Escherichia coli* and *Rothia mucilaginosa* when exposed to different aqueous ozone concentrations applied via an ultrasonic scaler for 5 through 6 s.

Bacterial Strain	(Aqueous Ozone) (mg/L)	Raw				Normalized to < 0.01 mg/L			
		Mean (Standard Deviation)	Minimum	Median	Maximum	Mean (Standard Deviation)	Minimum	Median	Maximum
*E. coli* MG1655	< 0.01∗	1,950.00 (593.55)	1,290.00	2,120.00	2,440.00	100.00 (30.44)	66.1538	108.7179	125.1282
	< 0.01†	700.00 (320.78)	420.00	630.00	1,050.00	100.00 (45.83)	60.0000	90.0000	150.0000
	0.52	57.33 (29.70)	24.00	67.00	81.00	2.94 (1.52)	1.2308	3.4359	4.1538
	0.78	2.27 (1.04)	1.10	2.60	3.10	0.32 (0.15)	0.1571	0.3714	0.4429
	1.12	5.83 (1.50)	4.40	5.70	7.40	0.30 (0.08)	0.2256	0.2923	0.3795
	1.25	0.05 (0.02)	0.03	0.04	0.07	0.01 (0.00)	0.0043	0.0057	0.0100
	1.60	0.41 (0.12)	0.31	0.38	0.54	0.02 (0.01)	0.0159	0.0195	0.0277
	2.65	0.02 (0.02)	0.00	0.02	0.03	0.00 (0.00)	0.0000	0.0029	0.0043
	3.76	0.00 (0.00)	0.00	0.00	0.00	0.00 (0.00)	0.0000	0.0000	0.0000
	4.53	0.00 (0.01)	0.00	0.00	0.01	0.00 (0.00)	0.0000	0.0000	0.0014
*R. mucilaginosa* 5762/67	< 0.01∗	4,866.67 (2,450.17)	2,400.00	4,900.00	7,300.00	100.00 (50.35)	49.3151	100.6849	150.0000
	< 0.01†	4,293.33 (244.40)	4,080.00	4,240.00	4,560.00	100.00 (5.69)	95.0311	98.7578	106.2112
	0.36	6,200.00 (3,377.87)	3,100.00	5,700.00	9,800.00	127.40 (69.41)	63.6986	117.1233	201.3699
	0.58	2,826.67 (1,172.40)	1,480.00	3,380.00	3,620.00	65.84 (27.31)	34.4720	78.7267	84.3168
	0.94	423.33 (210.32)	220.00	410.00	640.00	8.70 (4.32)	4.5205	8.4247	13.1507
	1.24	218.00 (38.94)	188.00	204.00	262.00	5.08 (0.91)	4.3789	4.7516	6.1025
	1.87	102.00 (86.61)	51.00	53.00	202.00	2.10 (1.78)	1.0479	1.0890	4.1507
	2.11	42.00 (3.61)	39.00	41.00	46.00	0.98 (0.08)	0.9084	0.9550	1.0714
	4.27	11.33 (14.47)	2.00	4.00	28.00	0.23 (0.30)	0.0411	0.0822	0.5753
	4.67	29.33 (5.69)	23.00	31.00	34.00	0.68 (0.13)	0.5357	0.7220	0.7919

∗,† Correspond to the negative control, sterile water applications for both runs 1∗ and 2†, respectively. Data are not combined due to separate normalization values.

For *R. mucilaginosa*, initial scaling with nonozonated water resulted in a recovery of 4,866.67 (SD 2,450.17 CFUs/mL) (Run 1) and 4,293.33 ± 244.40 CFUs/mL (Run 2). An ozone concentration of 0.36 mg/L showed no significant decrease in activity; however, increased concentrations did show a noticeable drop in the CFUs/mL recovered. An ozone concentration of 0.58 mg/L decreased the total recovered CFUs/mL by 34.16% ± 27.31%, and an ozone concentration of 4.67 mg/L decreased by 99.32% ± 0.13% (Figure 3B, Table).

## Discussion

### Efficacy of ozone on established biofilms

Ozone has long been used as a disinfecting agent, owing to both its efficacy against a broad range of organisms and viruses, and its rapid degradation.^24^, ^25^, ^26^, ^27^ It has been used widely in water sanitation and remediation,^28^ food safety^29^ and as a contact disinfectant on surfaces.^30^ By 2009, aqueous ozone and gaseous ozone have been shown to properly sterilize surgical and dental tools, expanding its use into the medical fields. In the light of the SARS-CoV-2 pandemic, ozone was once again evaluated for its ability to sterilize N95 respirators. It was found that, at concentrations effective for killing the influenza virus, the N95 respirators were properly sterilized of bacterial contaminants and their filtration rate or integrity was not affected.^31^ As such, the inclusion of aqueous ozone in an ultrasonic scaler reservoir has the potential for managing aerosolized microbes and possibly, airborne viral particles and may offer alternative sterilization procedures for dental and medical facilities to deal with future pandemics similar to the SARS-CoV-2 outbreak. A 2020 molecular modeling study by Tizaoui^32^ suggests that ozone would be an effective oxidant for SARS-CoV-2.

While exposure to gaseous ozone can induce oxidative stress in human respiratory tissue,^33^ aqueous ozone does not appear to cause damage to human oral cells or tissue.^34^, ^35^, ^36^ Short, controlled exposures have shown potential efficacy in treating infection and periodontal disease^25^^,^^34^^,^^37^ as well as aiding in pain management, wound healing, and cosmetic treatments.^25^^,^^36^ As such, use of aqueous ozone alongside ultrasonic scaling to remove dental plaque is not expected to cause any harm to the patient. Furthermore, ultrasonic scaling has long been shown to increase the bacterial load in the local atmosphere^1^, ^2^, ^3^ and has the potential for spreading pathogens through airborne droplets, direct inhalation, ocular membranes, or contact lens; this contamination poses increased risk to immunocompromised patients and dental personnel.^1^^,^^38^ Despite these risks, however, routine plaque control and debridement lead to an overall reduction in oral bacterial load and, as a result, a clinical improvement in periodontal disease.^39^

Previous data suggest that ozone treatment as an adjunct to regular scaling for patients with chronic periodontitis can aid in reducing overall bacterial load^37^; however, this interaction is reliant highly on individual methods of ozone preparation and application and cannot be relied on readily to act as antimicrobial treatment.^40^, ^41^ Likewise, ozone shows no benefit over the traditionally used sodium hypochlorite in decreasing bacterial load when used during root canal disinfection,^42^ but it does appear to aid in the overall wound healing after the procedure.^36^ Furthermore, application of gaseous ozone shows no significant antimicrobial activity when used during nonsurgical periodontal treatments.^43^ Our results suggest that the addition of ozone to the water supply of an ultrasonic scaler at concentrations capable of reducing the bacterial load (> 1.0 mg/L) of plaque biofilms is predicted to aid in the control of aerosolized microorganisms and viruses, though additional experimentation and validation will be performed in future studies. Our experimental setup used ultrapure Milli-Q water. The stability of aqueous ozone is known to depend on water source and purity.^44^ At a minimum, the use of deionized water should be considered in future studies and, if translated, into clinical use.

### Variation between *E. coli* and *R. mucilaginosa*

Much of the variation in the efficacy of ozone on the 2 model organisms we present likely can be attributed to the morphologic and physiological differences between the 2 organisms. It has been suggested previously that the thick peptidoglycan wall provides increased, but incomplete, resistance to ozone in gram-positive bacteria.^45^ Some gram-positive organisms also show increased resistance to ozone due to adaptation to the evolutionary pressure of consistent oxidant exposure, such as oxidizing disinfectants.^46^ Based on this, the *Rothia* species may exhibit resistance to ozone treatment as a by-product of resistance to oxidative stress in general. The *R. mucilaginosa* genome codes for several σ factors that are upregulated in response to oxidative stress.^13^ In addition, *R. mucilaginosa* can generate acetaldehyde from ethanol; increased levels of acetaldehyde are capable of inducing oxidative stress,^47^ suggesting that *R. mucilaginosa* has increased resistance to either acetaldehyde or the resultant oxidative stress it induces. This factor, combined with the thick peptidoglycan cell wall, may explain the variation in efficacy between the 2 organisms we studied.

Previous data examining the efficacy of ozone on the disruption of gram-positive biofilms suggest that ozone used in conjunction with another disruptive treatment results in significantly higher reduction in viable cells^48^; however, the effect of aqueous ozone alone on *R. mucilaginosa* biofilms was not examined during the course of our work. Furthermore, both species selected for our study are capable of aerobic respiration, and therefore, are more capable of oxidative stress responses than some anaerobic microorganisms commonly found in the oral cavity. These anaerobic species, as a result, would be expected to have a higher rate of mortality when exposed to aqueous ozone, as shown by Nagayoshi and colleagues^34^ using *Streptococcus* species. Although both *E. coli* and *R. mucilaginosa* have been shown to be susceptible to ozone and the biofilms produced by both organisms can be eliminated readily by the use of aqueous ozone dispensed through an ultrasonic scaler, further investigation into mixed culture biofilms and in vivo plaque disruption will still need to be explored to validate the procedure used in our study. Although our study thus establishes proof of concept for the combined use of aqueous ozone and ultrasonic scaling in the dental setting, there remain important limitations. For one, the tested strains are laboratory adapted domesticated strains and may have accumulated mutations making them more susceptible to the treatment modalities we tested. In addition, both strains serve only as representative gram-negative and gram-positive organisms and do not necessarily reflect the full range of physiological adaptations that may be found in a wider range of clinically relevant periodontal pathogens, either when grown in monoculture or in a mixed culture biofilm.

## Conclusion

Our study demonstrates that both gram-positive and gram-negative bacterial species are sensitive to the effects of aqueous ozone. Planktonic and biofilm-associated bacteria of both gram-negative *E. coli* and gram-positive *R. mucilaginosa* exhibited a decrease in viable colony-forming units following exposure to aqueous ozone. When combined with ultrasonic scaling biofilms formed by these organisms exhibited a further decrease in viable colony-forming units. Importantly, the working concentrations of aqueous ozone required for significant reduction in microbial load is readily achievable with commercially available equipment.
